# Active Humidity Control Chamber for Desorption Electrospray Ionization-Mass Spectrometry Imaging Applications

**DOI:** 10.1021/jasms.5c00111

**Published:** 2025-08-22

**Authors:** Hawkins S. Shepard, Robert L. G. Gottschalk, Jody C. May, John A. McLean

**Affiliations:** Department of Chemistry, Center for Innovative Technology, 5718 Vanderbilt University, Nashville, Tennessee 37235, United States

**Keywords:** Ambient ionization, DESI, relative humidity, environmental control, hygrostat/humidistat

## Abstract

Ambient ionization techniques enable mass spectrometry (MS) to expand into broader experimental contexts, although it is increasingly clear that results are influenced by the environmental conditions at the site of sampling. Desorption electrospray ionization (DESI), in particular, is affected by variations in relative humidity (RH) levels. Here we describe the design, development, and construction of an environmental control chassis that can actively modulate RH within ± 3% of user-defined set points across a broad humidity range (15%–70% RH). Preliminary characterization demonstrated differential analyte responses across a range of set points, with observed enhancement of leucine-enkephalin, sulfadimethoxine (negative mode), and maltose (positive mode) in response to increased humidity. The measurable differences in analyte signals across discrete humidity set points underscore the importance of environmental control in ambient ionization strategies. The humidity control system outlined here can be translated to other DESI platforms, with construction information provided herein.

## Graphical abstract

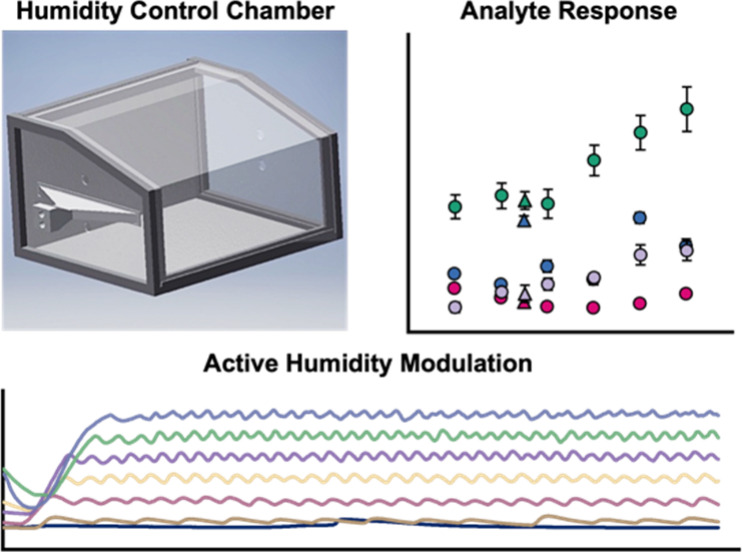

## Introduction

Ambient ionization techniques enable direct, *in situ* analysis of complex chemical systems with minimal sample handling requirements.^1–3^ One such ambient technique is DESI, which provides spatially resolved imaging capabilities in the native sample environments.^4–6^ DESI is utilized in forensic analysis,^7^ pharmaceutical screening,^8^ and microbial imaging.^9^ A growing body of work suggests that ambient MS results are strongly influenced by the local humidity present at the site of sampling.^10–12^ Ambient extractive and secondary ESI are highly dependent on humidity conditions.^13,14^ The variability of typical laboratory environments can limit DESI-MS reproducibility, particularly for extended duration MSI acquisitions. This environmental variability is confounded by the fact that the majority of DESI studies do not report specific laboratory conditions that would otherwise provide an analytical context for reproducing the work. However, no in-depth study has been undertaken to validate the role that environmental variability has on DESI-MS, in part because of a lack of strategies for controlling humidity during analysis. To address this, we have developed an active humidity control system for DESI, allowing for the real-time modulation of humidity levels during experiments.

This note outlines the construction and implementation of an environmental control chassis that allows for a considerable range of humidification set points (15%–70% RH) and incorporates a control loop that enables real-time adjustments of chamber humidity to within 3% of a user-defined set point. This system can modulate humidity concurrently with data acquisitions, without disrupting DESI sampling, and operate at constant humidity at time scales compatible with extended duration DESI-MSI. A panel of standards was analyzed at a number of humidity set points to assess the impact of laboratory environments on DESI-MSI acquisitions. Differential analyte responses were observed across various humidity conditions, suggesting that relative humidity within the ion source enclosure has a measurable effect on the DESI ionization efficiency and signal response.

## Experimental Methods

Experiments probing the effect of active humidity modulation implemented the “Fast-Pass” workflow.^15^ Briefly, solutions were prepared in equimolar mixtures at 50 μM in 50/50 methanol/water, deposited in 10 μL aliquots onto a 4 × 11 grid of raised polytetrafluoroethylene, and analyzed at discrete set points once evaporated. Standards and chemicals used and detailed acquisition parameters are outlined in Appendix S1. The source enclosure was modified by adding a gas inlet diffuser (flat conical geometry), exhaust port, and electrical feedthroughs for three humidity/temperature sensors. A listing of parts and manufacturers is provided in Table S1, with construction and electronic component information provided in Appendix S2. Interday replicates were obtained across 3 separate days, at 12 humidity conditions, from 180 individual spotted samples. Experiments were conducted in one ion polarity and then repeated in the opposite polarity within the same day.

## Results and Discussion

### Device Performance

The humidity modulation system was modified from a commercially available enclosure and incorporates a two-tier humidity control approach. Alternating “dry” and “wet” conditions achieve user-defined set points based off of sensor readbacks, operating on a control loop that allows real-time, automated adjustments. Gas flow is modulated by on/off solenoid control valves connected to a microcontroller actively monitoring temperature and humidity readings from three sensors, introducing more dry carrier gas when RH readback rises above the user-defined set point and more humidified carrier gas when readback falls below the set point. System schematics, wiring overviews, component information, and control system code developed in-house can be found in Figure S1, Figure S2, Table S1, and Appendix S3, respectively. Gas introduction is distributed within the enclosure via a 3-D printed gas inlet, diffuser nozzle, and exhaust port (Figures S3, S4, and S5), with the design of these components guided by computational fluid dynamic modeling (Figure S6) to ensure optimal airflow dispersion while minimizing gas flows at the DESI spray and ion transfer regions. Printing instructions for the inlet, diffuser nozzle, and sensor housings/exhaust ports can be found in the Supporting Information.

The “dry” condition introduces unmodified compressed air sourced from a laboratory gas line (>25 psi). The “wet” condition passes the laboratory air through a bubbler system, wherein gas flows into a media bottle containing high purity water before the chamber. A single nozzle is used to deliver the carrier gas for both conditions. A carrier gas flow pressure of 4 psi was found to be optimal for sensor response without disrupting DESI-MS signal. Three individual sensors are used to record both the temperatures and relative humidity values at different locations (Figure 1A). Because the readback of sensor 2 is affected by heat radiating from the source desolvation block, only the readback from sensors 1 and 3 is used for real-time modulation, with the readback average dictating the humidity condition applied. No modulation is applied when average values fall within a cutoff threshold of ± 3% RH. This threshold encompasses the ± 2% RH tolerance of the sensor, minimizing unnecessary gas flow.

**Figure 1:**
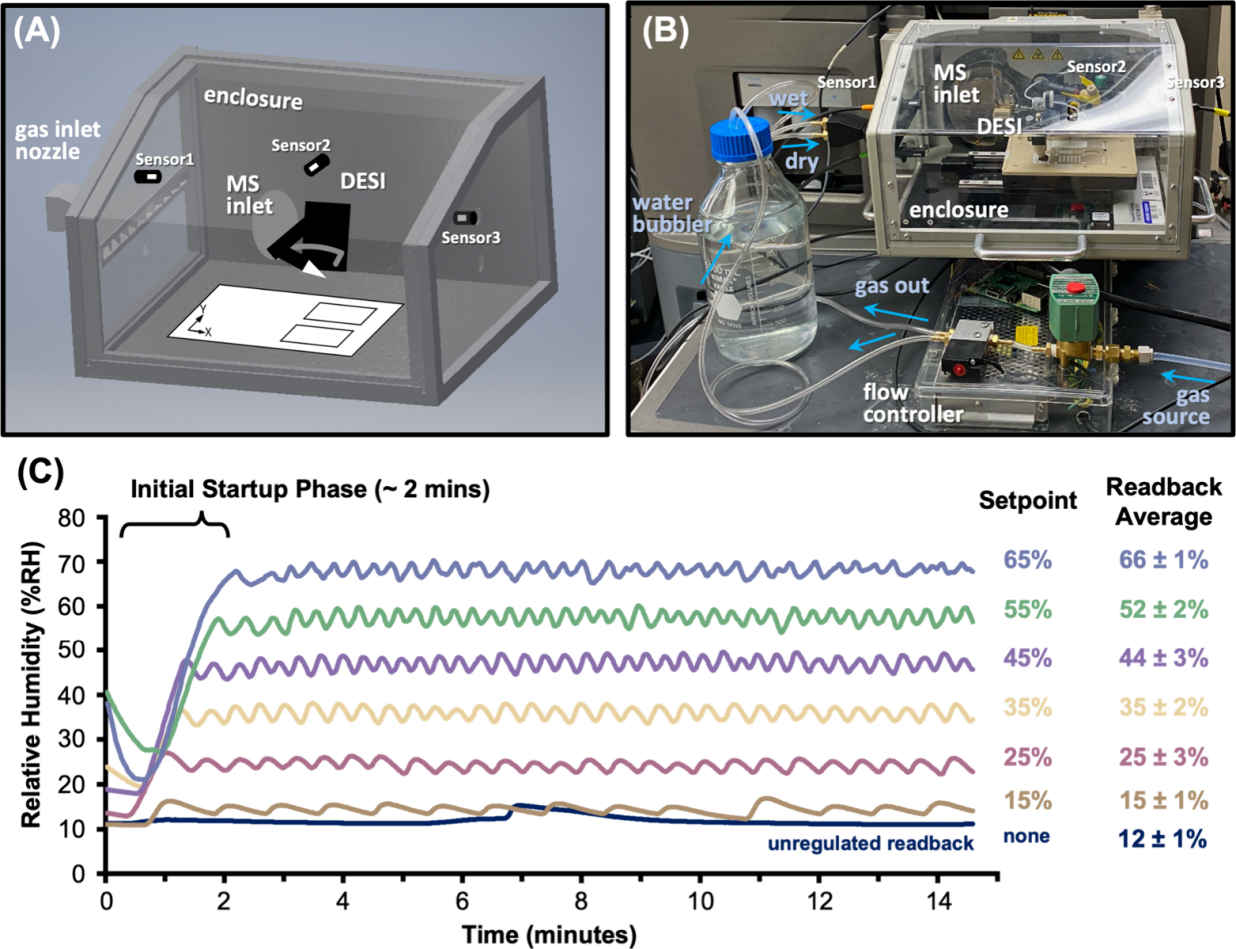
Design and performance of the humidity control system. (A) Digital rendering of the enclosure, highlighting the locations and orientations of climate sensors 1, 2, and 3. (B) Photograph of the system with important functional components labeled. Sensors 1, 2, and 3 are dual temperature and humidity probes providing active readbacks. The water bubbler provides the humidity source for the “wet” condition, whereas the “dry” condition bypasses the bubbler. The microcontroller and solenoid valves are contained in an acrylic flow controller box. Gas flow directions indicated by arrows. (C) Humidity readback data across 15 min for various user-defined set points. Unregulated readback corresponds to no active humidity control.

The enclosure and controller assembly are pictured in Figure 1B. Enclosure performance (Figure 1C) indicates an initial response delay (<2 min) preceding real-time humidity modulation to establish a steady-state. For each set point, the system maintains desired levels within the designated threshold (±3% RH) for the monitored duration (15 min). Initial startup duration lengthens at higher set points due to larger differences between the set point and ambient humidity. For all set points, the readback value was maintained within 3% of the set point and deviating 3% or less, indicating the system can achieve accurate and stable RH control. The largest deviation was observed at midrange set points (25% and 45% RH). The smallest was measured at more extreme set points (15% and 65% RH). The high deviations at intermediate humidities could be due to longer response times associated with switching between conditions, whereas more extreme settings require only one conditions to be applied.

### Effect of Humidity on Analyte Signal

To examine the effects of humidity modulation on ionization efficiency and signal response, six chemical standards representing different chemical classes were analyzed across discrete set points in positive and negative polarities (Figure 2). Interday replicates (3 days, n = 15 for leucine-enkephalin; n = 5 for all other analytes) were acquired using a randomized sequence of humidity set points to ensure that observed trends were not biased through systematic fluctuations or temporal drifts in instrument response. Acquisitions with no humidity control provide a basis of comparison and represent an average ambient laboratory humidity level of 35% RH. In comparing integrated total signal intensities and integrated intensities of individual analytes for these single-raster acquisitions, measurable variations in analyte responses are observed in both ion polarities. The average interday measurements obtained at each RH value are reproducible to within 10% of the signal average, measured across three separate days. Statistical significance for signal comparisons reported was validated by one-way ANOVA for each of the individual analytes (p-value <0.01).

**Figure 2:**
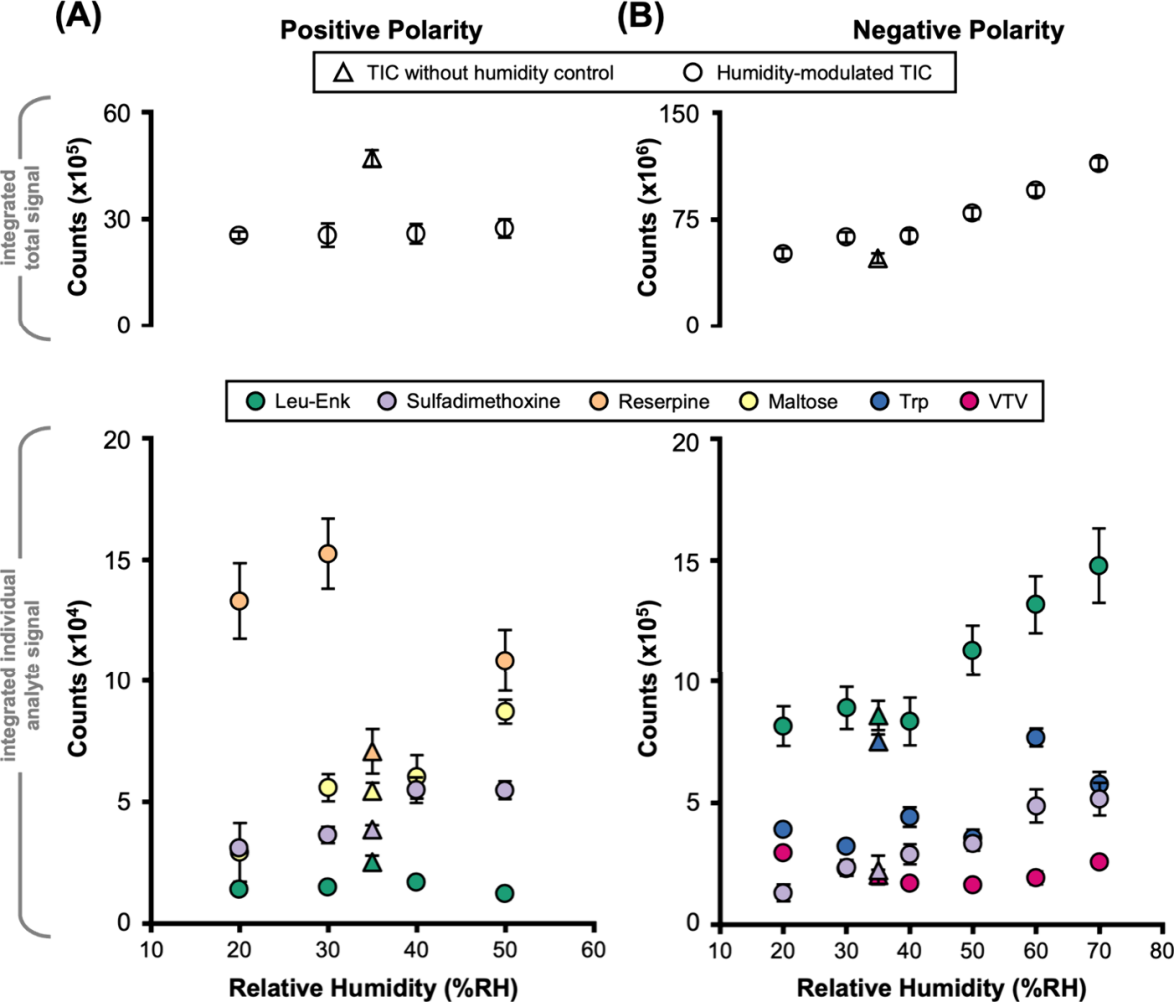
Effect of humidity modulation on various analytes in (A) positive and (B) negative polarity. Differential response is observed for total signal (top) as well as leucine-enkephalin internal standard and selected analytes (bottom). Plot data is averaged across replicates (*n* = 15 interday for integrated total signal and leucine-enkephalin, *n* = 5 for all other analytes). Error bars represent standard error.

For positive polarity (Figure 2A), the total signal is higher for the uncontrolled humidity condition (∼35% RH) versus the controlled conditions; however, analyte-specific results indicate that this is not general, with some analytes exhibiting higher signal at the higher humidity levels. Specifically, sulfadimethoxine ([M + H]^+^, *m*/*z* 311.08) and maltose ([M + Na]^+^, *m*/*z* 365.11) exhibit a notable increase in the signal at higher humidities. In contrast, the two highest mass analytes investigated, leucine enkephalin ([M + H]^+^, *m*/*z* 556.27) and reserpine ([M + H]^+^, *m*/*z* 609.28), exhibit either no or inconclusive changes in the positive ion signal with respect to humidity. In all cases, positive ion signal either remains constant or increases with increasing humidity.

For the negative ion mode (Figure 2B), the total signal increases with higher humidity, and most of the analyte-specific XICs also reflect this trend. Both leucine enkephalin ([M−H]^−^, *m*/*z* 554.26) and sulfadimethoxine ([M−H]^−^, *m*/*z* 309.07) exhibit more than twice the signal at high RH in comparison to low RH, with less consistent trends observed for tryptophan ([M−H]^−^, *m*/*z* 203.08) and valine-tyrosine-valine tripeptide ([M−H]^−^, *m*/*z* 378.20). Whereas the varied signal responses at different RH conditions discussed here are statistically significant, we acknowledge that the relatively small pool of samples investigated potentially limits the generalizability of these results to other analyte classes and to more complex samples, such as tissue sections. Nevertheless, the data obtained from both ion modes suggest that there are measurable analytical benefits to operating DESI under high humidity conditions. While this investigation focused on the effects of active humidity modulation on the analyte signal, preliminary experiments have indicated that this system is able to maintain humidity set points without any signal loss over the course of extended duration experiments (Figure S7).

## Conclusions

Performance evaluation of the DESI-MS environmental control system demonstrates precise control of humidity (within 3%) across a wide range of RH set points (15–65%). Preliminary characterization across various user-defined humidity set points found differential analyte responses and indicate measurable analytical benefits for operating DESI under controlled humidity conditions. However, observed trends are difficult to generalize, suggesting that optimal conditions are likely system-dependent. These findings further highlight the importance of environmental control in ambient ionization strategies, suggesting that tailored humidity modulation could enhance the DESI performance. Of note, aspects of the humidification system described here should be readily transferrable to other commercially available DESI platforms incorporating a source enclosure (e.g., DESI XS) with only minimal alterations. Future work will focus on further characterizing the impacts of humidity control on specific DESI performance metrics within the context of extended duration MSI applications.

## Supplementary Material





## Notes

Schematics, fluid dynamics simulation results, extended duration DESI-MSI results, a summary of components with vendor sources, appendices detailing DESI-MSI parameters, construction information, and C++ code; visual representations of 3-D printed components (PDF)
3D printer instruction files (ZIP)

## Abbreviations

DESI-MSI: desorption electrospray ionization mass spectrometry imaging
*m*/*z*: mass-to-charge ratio
RH: relative humidity
