# Supplementary material for: Active Humidity Control Chamber for Desorption Electrospray Ionization-Mass Spectrometry Imaging Applications
Source: J Am Soc Mass Spectrom. 2025 Aug 22;36(9):1995–9. doi: 10.1021/jasms.5c00111 (PMC12412154; doi:10.1021/jasms.5c00111)
Supplement: Supplementary file 1 [file js5c00111_si_001.pdf]

Supporting Information

Active Humidity Control Chamber for Desorption Electrospray Ionization-Mass Spectrometry Imaging Applications

Hawkins S. Shepard, Robert L. G. Gottschalk, Jody C. May, John A. McLean*

Center for Innovative Technology, Department of Chemistry, Institute of Chemical Biology, Institute for Integrative Biosystems Research and Education, Vanderbilt-Ingram Cancer Center, Vanderbilt University, Nashville, Tennessee 37235 United States

* Corresponding Author Email: john.a.mclean@vanderbilt.edu

SI Contents:

Figure S1 – Environmental Control Chamber System Schematics …….…..…...….……………….. S2
Figure S2 – Overview of Electronic System Wiring ………………..….…...…..…...…………… S3
Figure S3 – Mechanical Drawing of Sensor Holder and Exhaust Port Component.....……………. S4
Figure S4 – Mechanical Drawing of Gas Inlet ………………………………….....…………… S5
Figure S4 – Mechanical Drawing of Gas Diffuser ……….………………………....…………….. S6
Figure S6 – Fluid Dynamic Modeling for Nozzle Design ………..…...…...…..…...……………. S7
Figure S7 – Preliminary Long-Format DESI-MSI Implementing Humidity Control ..…………….S8
Table S1 – Humidity Control System Components…...………....…….…….….…….……………S9
Appendix S1 – DESI Acquisition Parameters and Data Processing ……..……...….…………….S10
Appendix S2 – Construction Information for Environmental Control Chamber ……..……………S11
Appendix S3 – C++ Code for Control System Feedback Loop…..………..……..…..…………….S12

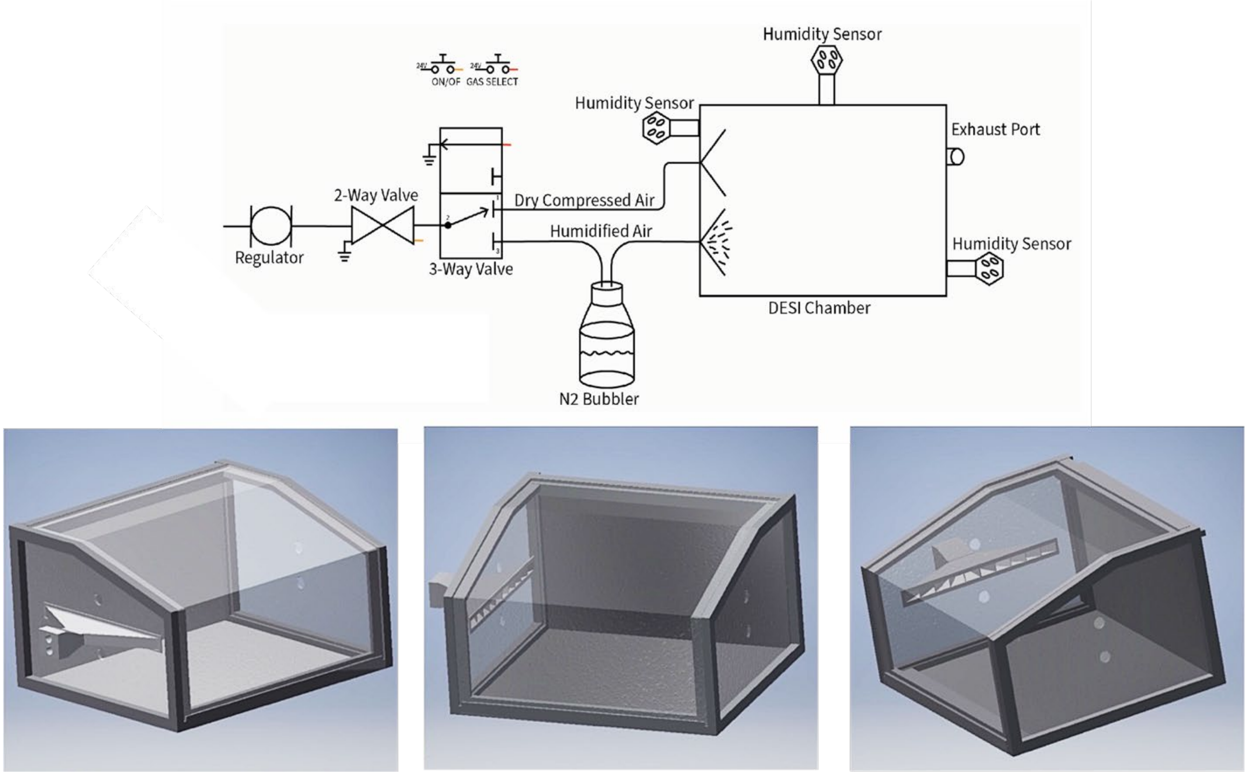

Figure S1. Environmental control chamber system schematics and additional digital renderings showing system orientations.

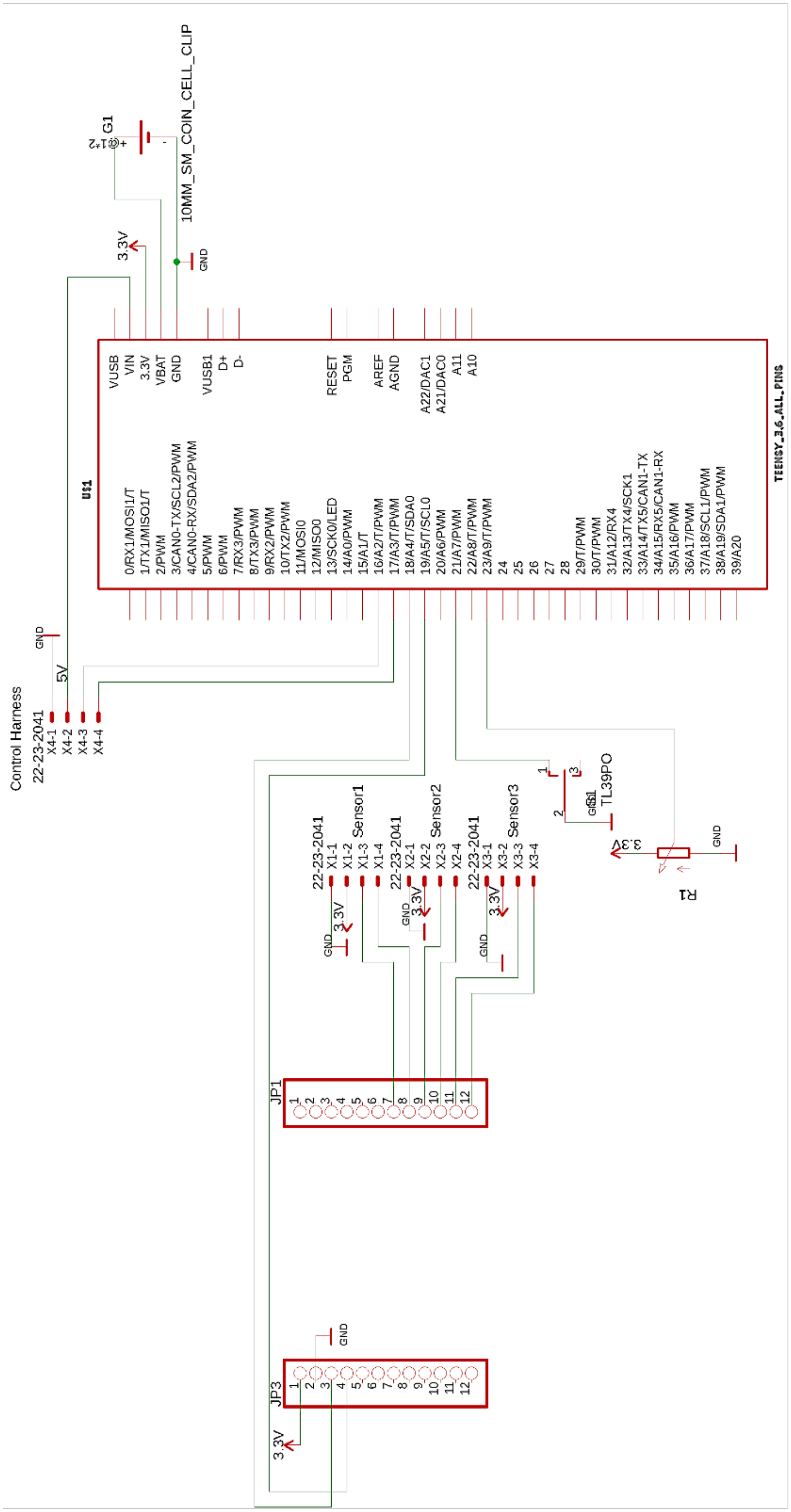

Figure S2. Overview of Electronic System Wiring.

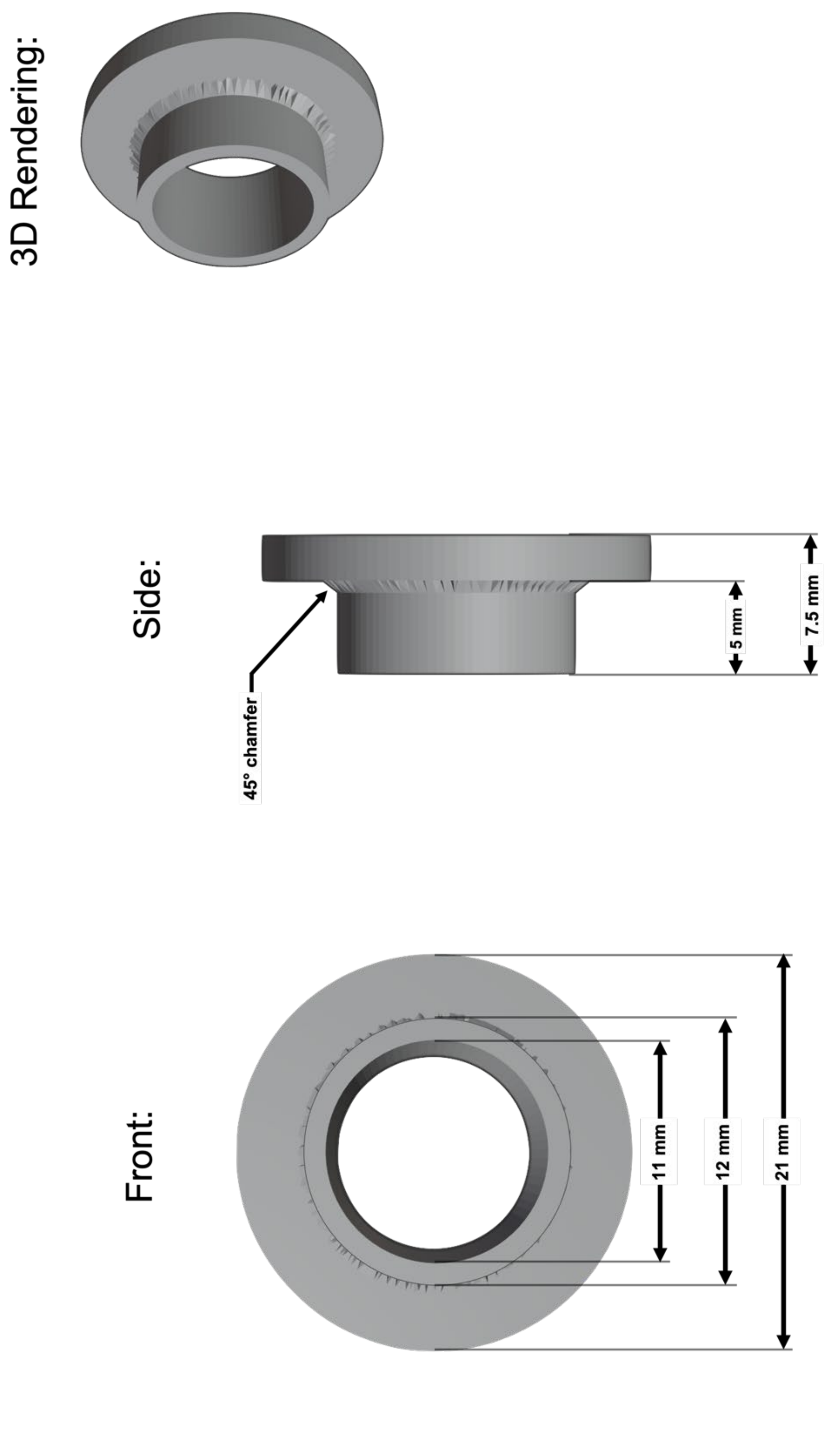

Figure S3. Mechanical Drawing of Sensor Holder and Exhaust Port Component.

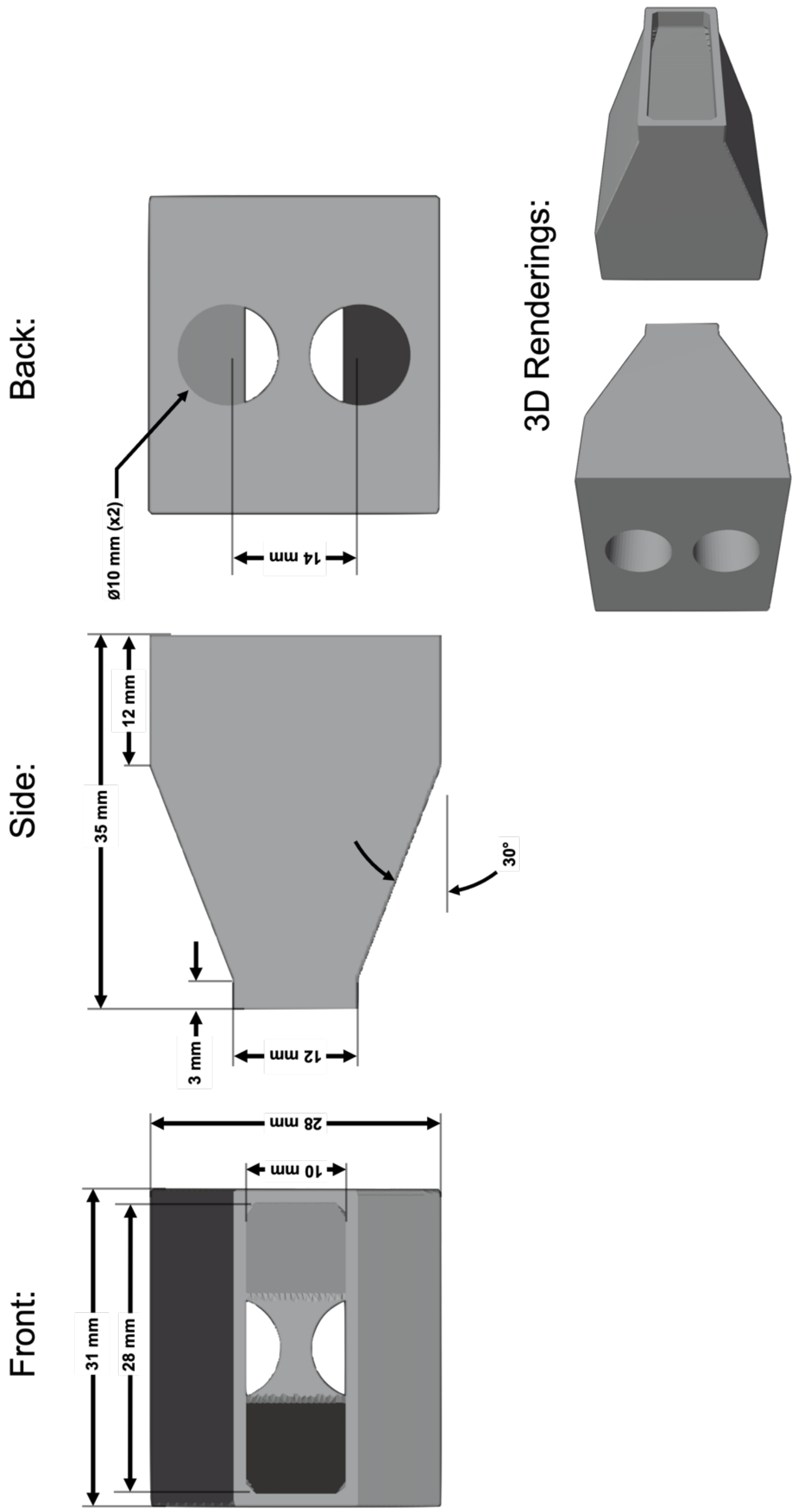

Figure S4. Mechanical Drawing of Gas Inlet.

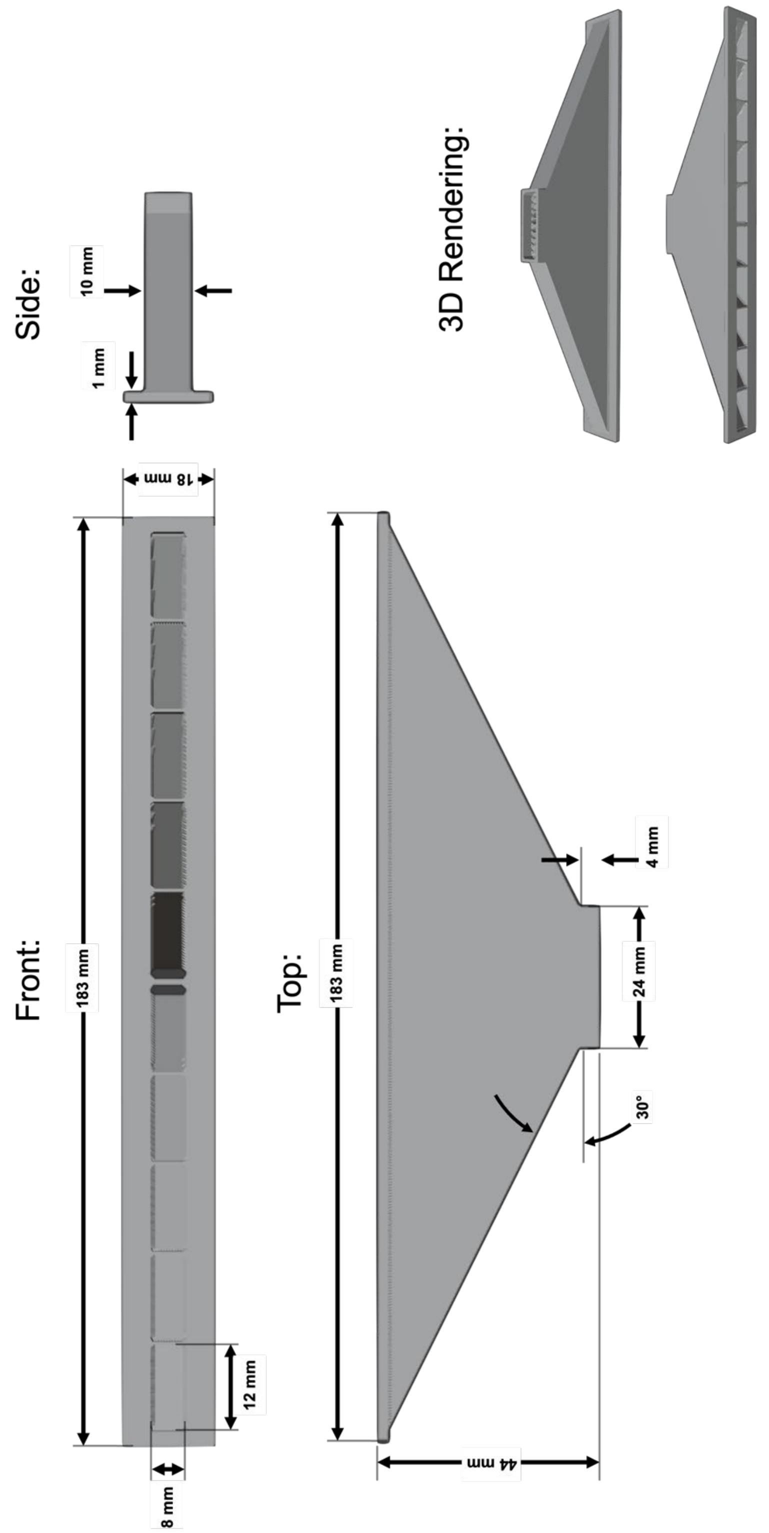

Figure S5. Mechanical Drawing of Gas Diffuser.

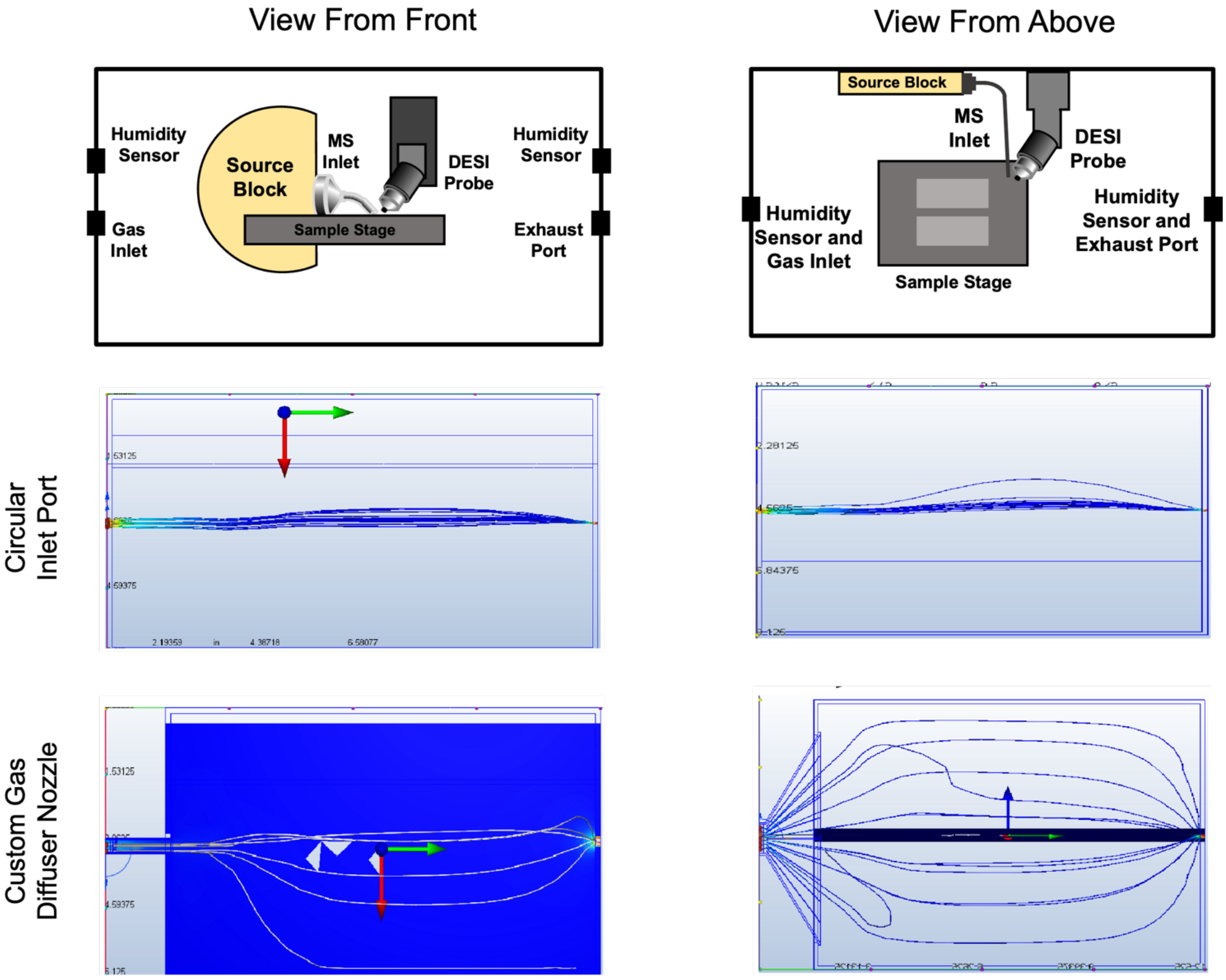

Figure S6. Computational fluid dynamic modeling results used to inform the optimal design and position of the 3D-printed nozzle and gas carrier distribution system to ensure sufficient airflow dispersion with minimal perturbation of gas flow at the site of DESI sampling.

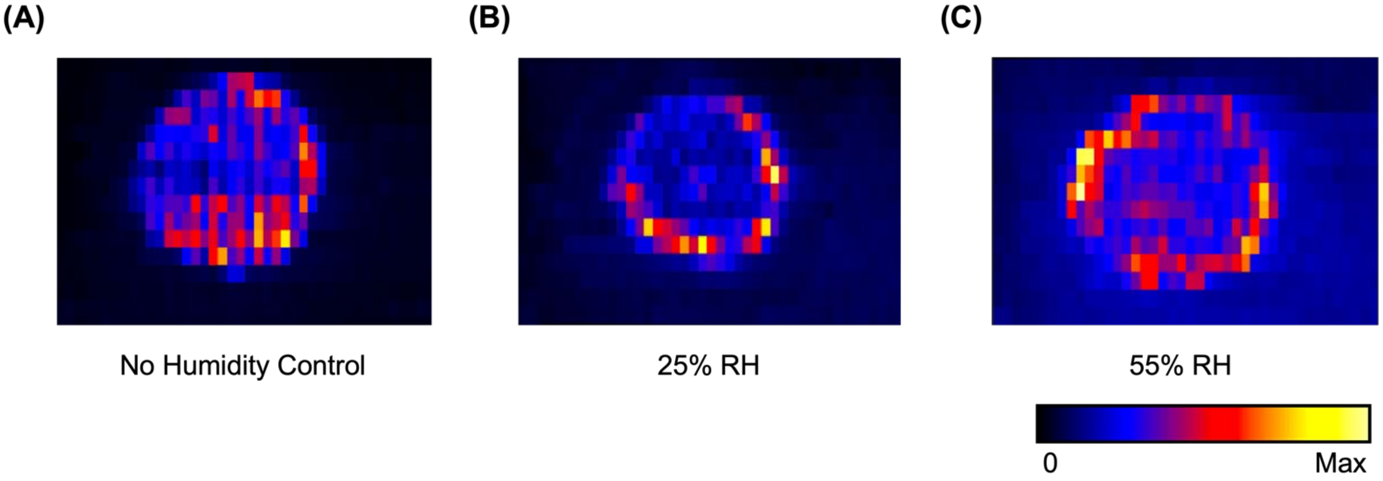

Figure S7. A long-format (~20 min) DESI-MSI experiment of a single sample spot using active humidity control to modulate relative humidity (RH) within the sample chamber. Ion heat maps represent data corresponding to tetradecanoic acid (*m/z* 227.20) manually spotted onto raised polytetrafluoroethylene spots (3 mm diameter). The humidity conditions applied were (**A**) no active control, (**B**) 25% RH, and (**C**) 55% RH. The regulated conditions show analyte localization along the perimeter of the dried sample spot (the so-called "coffee ring" effect), due to analyte migration and concentration along the liquid-substrate interface during the sample spot drying process. No spatial normalization has been applied, but pixel intensities have been normalized across acquisitions.

Table S1. Humidity control system components, vendor sources, and part numbers. A description of the use for each component is provided in the last column.

Component	Quantity	Vendor	Part No.	Use
12V DC Brass Solenoid Valve	1	Grainger	6WTT7	Switching carrier gas flow on/off
T9602-3-D-1 Humidity and Temperature Probes	3	Mouser	527-T9602-3-D-1	Dual humidity and temperature readback
24V DC Solenoid Air Control Valve	1	Grainger	6JJ53	Switching carrier gas flow between humidification conditions
Adafruit TCA9548A 1-to-8 I2C Multiplexer Breakout	1	Mouser	485-2717	Combining sensors to same I2C
150W Ultra-High Peak Power Supply	1	Mouser	709-HRP-150N3-36	Power supply for all electronic components
24V Relay Breakout Board	2	Mouser	992-MR-BUSIO-RELAY	Switching relays for solenoids
Barbed Fittings	8	Grainger	6AFP4	Solenoid connections
Carrier Gas Tubing	1	Grainger	55YG36	Tubing for direction of carrier gas stream
Teensy 3.6 Microcontroller	1	Mouser	485-3266	Microcontroller for control system feedback loop
I2C Display	1	Mouser	426-DFR0648	Screen display for microcontroler

Appendix S1 – DESI Acquisition Parameters and Data Processing.

High purity (Optima Grade) methanol, water, and acetonitrile were purchased from Fisher Scientific. Analytical standards of leucine-enkephalin, valine-tyrosine-valine (VTV), tryptophan, maltose monohydrate, sulfadimethoxine, and reserpine were obtained from MilliporeSigma. All chemicals were used as received. All experiments were carried out on a Waters Synapt G2-S mass spectrometer (Waters Corporation) incorporating a legacy DESI-MSI probe (Prosolia Inc). Experiments were conducted with the mass spectrometer sequentially operating in both negative and positive ion modes. Relevant source parameters include: ± 5 kV capillary voltage, 150°C source temperature, 0.45 bar of N_2 gas flow, a 70° sprayer angle, a 0.7 mm ablation diameter, a 125 µm/s raster rate, and an x, y, z positional orientation relative to the MS inlet of -2, +2, +2.75, respectively. The DESI ionization solvent used consisted of 90/10 acetonitrile/water, 0.2 ng/µL leucine enkephalin, and 0.1% formic acid for positive ion mode or 0.1% ammonium hydroxide for negative ion mode. Pixel dimensions for Fast-Pass experiments were 50x1000 µm. Preliminary long-format imaging experiments were carried out using the same source parameters but with pixel dimensions of 500x200 µm. The MS was mass calibrated with sodium formate salt clusters to a 95% confidence band with a mass range of *m/z* 50-1200. Lock mass adjustments were performed in HDImaging using the leucine enkephalin present in the spray solution. Processed .txt files were imported into Microsoft Excel for further data treatment, including spatial normalization. Signals associated with chemical standards were identified based on an accurate mass threshold of 10 ppm.

Appendix S2 – Construction Information for Environmental Control System.

Modification of the commercial enclosure into an environmental control chassis involved laser cutting sheets of 1/8 inch clear acrylic to match the dimensions of the acrylic side panels of the commercial enclosure. Specific dimensions will depend on commercial enclosure being modified. Additional cuts were made to accommodate the gas diffusion nozzle and the sensor holders, with these pieces being glued in place using cyanoacrylate glue prior to chamber assembly. 3-D printing instructions are included as .zip files and provided along with the SI. An enclosure for the electronic components was constructed out of a small acrylic case, with the power supply, the relay board, and the solenoid valves being affixed within case. The relay board was wired to the power supply, and a wiring harness was built with a 1x4 female DuPont connector to provide relay input and power to the control system. The Teensy 3.6 was soldered to header pins and the accompanying socket was placed on a breadboard with the accompanying connections to the TCA9548, I2C display, and coin-cell battery. The TCA9548 I2C multiplexer was connected directly to the humidity sensors and enabled multiple sensors with the same default address to exist on the same I2C communication bus. An overview of the wiring schematics is provided in **Figure S2**. While a custom printed circuit board (PCB) was not constructed for this project, the provided schematic imported into EAGLE PCB designer would enable a custom breakout board to be manufactured in-lieu of wired connections between the control and relay boards.

Appendix S3 – C++ Code for Control System Feedback Loop.

```
/*Name: DESI_Control_Interface_1
 *Description: DESI-MS Humidity Control script using TEENSY 3.6, TCA9548, MICRO-SD card, & 3 x Amphenol T9602 Humidity
Sensors.
 *-------------See DESI
 *Date: 3/31/2022
 */
#include <Wire.h>
#include "T9602.h"
#include <TimeLib.h>
#include <Bounce.h>
#include <SD.h>
#include <string.h>
#include <math.h>
#include "TeensyTimerInterrupt.h"
#include <Adafruit_GFX.h>
#include <Adafruit_SSD1306.h>
/*
 * Addresses & Variables used for IO & Digital Operations
 */
// Define OLED Display
#define SCREEN_WIDTH 128 // OLED display width, in pixels
#define SCREEN_HEIGHT 32 // OLED display height, in pixels
#define OLED_RESET     4 // Reset pin # (or -1 if sharing Arduino reset pin)
#define SCREEN_ADDRESS 0x3C ///< See datasheet for Address; 0x3D for 128x64, 0x3C for 128x32

Adafruit_SSD1306 display(SCREEN_WIDTH, SCREEN_HEIGHT, &Wire, OLED_RESET);

//TCA9548 addresses used for communication & port selection
#define I2cSelect 0x70 // I2C channel selector address on the bus
#define Sense1 0b00100000  //Sense1 probe
#define Sense2 0b01000000  //Sense2 probe
#define Sense3 0b10000000 //Sense3 probe

//Pin definitions
#define R1 17 //Relay 1
#define R2 16 //Relay 2
#define chipSelect BUILTIN_SDCARD //Used to select SPI ports for onboard SD_CARD on Teensy 3.6
#define Ind 13 //Indication LED connected to pin 13
#define Pot A9 //Defines potentiometer input

//Button Definitions
#define BU1 29 //Button 1
#define BU2 28 //Button 2
#define BU3 21 //Button 3: On/OFF switch linked to active Mode
//Bounce constants, tuned to 5 millisecond delay period after button-press (measured with oscilloscope)
```

```
Bounce UP = Bounce(BU1,100);
Bounce DOWN = Bounce(BU2,100);

//Global Variables
float CurrentVals[7]; //[(Humidity)S1,S2,S3,(Temp)S1,S2,S3,HAVG], Data Structure for the sensor variables
float HumiditySetpoint = 35; //Set by the user in idle and then used in control loop during active
float SystemStatus = 0; //idle = 0, Active = 1
unsigned long Freq = 1000000; //Active Interval (in μs)
unsigned long ScreenRefresh = 500; //Screen refresh (in ms)
float Tolerance = 3; //Sensor Tolerance, absolute value of Sensor-Humidity setpoint is used for decision loop
String Header[9]={"Time","H1","H2","H3","T1","T2","T3","SystemStatus","HumiditySetpoint"};
char Filename[28]={0}; //Character Holder for filename generated by Filer()
float SystemPreferences[2]={SystemStatus,HumiditySetpoint}; //Loads Status registers into variable array
unsigned long FlagPrint = 0; //Print Marker: used w/ScreenRefresh
bool FlagWrite = 0; //Write Marker, locks out display print
unsigned long ActiveFlag = 0; //Active Marker: Used in active state to avoid relay wear
float vals = 0; //Used for potentiometer calculations

//Class Definitions for Custom Libraries
T9602 Sense; //Defines T9602 class for the accompanying library
IntervalTimer MyTime;
/*
 * User Controls and Outputs, used in the alteration of control loop variables
 */
//used to write Potentiometer values
void GetPot(){
  vals = analogRead(Pot); //reads potentiometer
  float intermediate; //Intermediate value holder
  intermediate = 100*(vals/1023); //Performs humidity calculation based on 10-bit ADC
  HumiditySetpoint = (int) intermediate; //Casts float -> int
}
//LED digital write selector: used to control the different LED OUTPUTS avaialble for status indication
void Status(uint8_t Channel, bool Signal){
  digitalWrite(Channel,Signal);
}
void testdrawtriangle(void) {
  display.clearDisplay();
  for(int16_t i=0; i<max(display.width(),display.height())/2; i+=5) {
    display.drawTriangle(
      display.width()/2  , display.height()/2-i,
      display.width()/2-i, display.height()/2+i,
      display.width()/2+i, display.height()/2+i, SSD1306_WHITE);
    display.display();
    delay(1);
  }
  delay(2000);
}
void PrintAvg(void) {
```

```
  display.clearDisplay();
  display.setTextSize(2); // Draw 2X-scale text
  display.setTextColor(SSD1306_WHITE);
  display.setCursor(10, 0);
  display.print(CurrentVals[6]);
  display.setTextSize(1);
  display.setCursor(10, 20);
  display.print(HumiditySetpoint);
  display.display(); // Show initial text
}
void Message(int state){
  switch(state){
     case 1:
      display.clearDisplay();
      display.setTextSize(1); // Draw 2X-scale text
      display.setTextColor(SSD1306_WHITE);
      display.setCursor(10, 0);
      display.print("Initializing Card");
      display.display();
      break;
    case 2:
      display.clearDisplay();
      display.setTextSize(1); // Draw 2X-scale text
      display.setTextColor(SSD1306_WHITE);
      display.setCursor(10, 0);
      display.print("Card Initialization Error");
      display.display();
      break;
    case 3:
      display.clearDisplay();
      display.setTextSize(1); // Draw 2X-scale text
      display.setTextColor(SSD1306_WHITE);
      display.setCursor(10, 0);
      display.print("Card Initialized");
      display.display();
      break;
    case 4:
      display.clearDisplay();
      display.setTextSize(1); // Draw 2X-scale text
      display.setTextColor(SSD1306_WHITE);
      display.setCursor(10, 0);
      display.print("Flip Switch to Off");
      display.display();
      break;
    case 5:
      display.clearDisplay();
      display.setTextSize(1); // Draw 2X-scale text
      display.setTextColor(SSD1306_WHITE);
```

```
      display.setCursor(25, 0);
      display.print("Write Error");
      display.display();
      Serial.print("somethings up");
      digitalWrite(Ind,HIGH);
      break;
  }
}
/*
 * Operations related to the reading of the sensors connected to Teensy & the RTC
 */
//Loop responsible for reading the 3 sensor inputs and returning the data by writing to the CurrentVals array
void ReadSeries(){
  int indexr[3] = {Sense1,Sense2,Sense3}; //Internal loop variables for sensor channels for I2c Switcher
  int tempr_Offset = 3; //used to displace the temperature indices in the CurrentVals Array
  for (int x = 0;x<3;x++){
      Wire.beginTransmission(I2cSelect); //selects TCA9548 default I2C address
      Wire.write(indexr[x]); //writes the function's pre-selected address
      Wire.endTransmission(); //terminates I2C for sensor reading
      Sense.updateMeasurements(); //T9602 reading
      CurrentVals[x] = Sense.getHumidity(); //Humidity Storage
      CurrentVals[x+tempr_Offset] = Sense.getTemperature(); //Temperature Storage
      delay(1);//Help with I2C delays
   CurrentVals[6] = (CurrentVals[0]+CurrentVals[2])/2; //Taking average of left and right sensor
  }
}
void Transfer(){
  SystemStatus = SystemStatus * 1;
  HumiditySetpoint = HumiditySetpoint * 1;
}
time_t getTeensy3Time(){
  return Teensy3Clock.get();// Used to return the RTC value in the Time in seconds since 1/1/1970
}
/*
 * SD CARD Operation functions
 */

// Date conversion for file naming
String date(){
  String dAy = String(day()); //convert day to string
  String mOnth = String(month()); //convert month to string
  String yEar = String(year()); //convert year to string
  String Date = dAy + "_" + mOnth + "_" + yEar; //string of entire date
  return Date;
}
//Time conversion for file naming (unix compatible characters)
String stimer(){ //Used for writing file name (since using dashes instead of colons)
  String HR = String(hour()); //int to string
```

```
  String MIN = String(minute());
  String SEC = String(second());
  String STIMER = HR +"-"+ MIN +"-"+ SEC; //collect time for date
  return STIMER;
}
//Time convesion for data entry in CSV
String timer(){ //Used for writing csv data entries
  String HR = String(hour()); //int to string
  String MIN = String(minute());
  String SEC = String(second());
  String TIMER = HR +":"+ MIN +":"+ SEC; //collect time for date
  return TIMER;
}
//Ascertains the RTC at turn on and writes the date & time to corresponding trial csv
void FILER(){ //Writes the file name with the initial date and time (operating system safe)
  String TIME = stimer(); //returns string happy timer without colons
  String DATE = date(); //same function
  String Filer = DATE + "(" + TIME +")"+ ".csv";
  Filer.toCharArray(Filename,28); //used to convert string to character array for SD.open fxn
  File dFile = SD.open(Filename, FILE_WRITE); // collects time, turns to array, and writes csv
  if (dFile) {
    for(int z =0; z<8; z++){
      dFile.print(Header[z]); // writing header for csv (found in array above)
      dFile.print(",");
    }
    dFile.println(Header[8]);
  }
  else{
    Message(5); //Error if sd card does not establish new file name for trial
  }
}

//Used to write all 11 columns of data as individual rows (Time,H1,H2,H3,T1,T2,T3,Status,HumiditySetpoint,OnTime,Offtime)
void Writer(){
  FlagWrite = 1;
  ReadSeries(); //Reads the value from all 3 sensors
  Transfer();
  float SystemStatus1 = SystemStatus;
  float HumiditySetpoint1 = HumiditySetpoint;
  float SystemPreferences1[2]={SystemStatus1,HumiditySetpoint1}; //Loads Status registers into variable array
  char TIMEs[10] = {0}; //Time storage
  String TIM = timer(); //Acquires time
  String datas[9]= {0}; //used for packaging each row of the csv
  TIM.toCharArray(TIMEs,10); //converts first cell from String to char time
  datas[0]=TIMEs; //loads into data storage
  for (int x=0; x<6; x++){
    char dats[10];
    sprintf(dats,"%f",CurrentVals[x]); //converts floats into char
```

```
    datas[x+1]=dats; //loads char into the array
  }//prints to entry 7
  for (int x =0;x<2;x++){
    char paste[6] = {0};
    sprintf(paste,"%f",SystemPreferences1[x]); //converts System preferences/conditions to values for the csv print array
    datas[x+7]=paste; //pasting control data into data container
  }//prints to entry 11
  File dFile = SD.open(Filename,FILE_WRITE); //writing to data file after data conversion
  if (dFile) {
    for(int z =0; z<8; z++){
      dFile.print(datas[z]); // writing header for csv (found in array above)
      dFile.print(",");
    }
      dFile.println(datas[8]);
      dFile.close(); //closing line after the write is finished
   }
  else{
    Message(5);
  }
  PrintAvg(); //Calls printing program
}
/*
 * Control Loop Operations
 */
//Function used to raise the humidty of the test chamber
 void HumidOn(){
  digitalWrite(R2,HIGH); //Switches the directional valve to the humidity position
  delay(10); //Small 10 ms delay used between actuatuon of directional valve and on/off
  digitalWrite(R1,HIGH); //Solenoid on/off valve
 }

//Function used to lower the humidity of the test chamber
void DeHumidOn(){
  digitalWrite(R1,HIGH); //Solenoid switches on to pulse dry air
 }
//Function used to clear the active phase from both humidification and dehumidification cycles
void ActiveOff(){
  digitalWrite(R1,LOW); //Switches the on/off solenoid to the off position
  digitalWrite(R2,LOW); //Switches the directional solenoid off
}
/*
 * INIT CODE
 */
void setup() {
  Wire.begin(); //initates I2C port for the Switcher
  delay(2000);//Used to prevent multiple files from being written
  //Teensy Control outputs (Status LED & Relays)
```

```
  pinMode(R1,OUTPUT); //Relay1 output, corresponding to on/off valve
  pinMode(R2,OUTPUT); //Relay2 output, corresponding to directional valve (default dry)
  pinMode(Ind,OUTPUT); //onboard LED used for debugging purposes
  //Push-button pull-up resistors
  pinMode(BU1,INPUT_PULLUP);
  pinMode(BU2,INPUT_PULLUP);
  pinMode(BU3,INPUT_PULLUP);
  Serial.begin(9600);
  Serial.print("hellO");
  //Protocol & Bus initialization
  setSyncProvider(getTeensy3Time); //Sets the RTC provider as the onboard RTC on the Teensy 3.6 (req. -> coin-cell battery)
  // SSD1306_SWITCHCAPVCC = generate display voltage from 3.3V internally
  if(!display.begin(SSD1306_SWITCHCAPVCC, SCREEN_ADDRESS)) {
    for(;;); // Don't proceed, loop forever
  }
  // Clear the buffer
  display.clearDisplay();
  testdrawtriangle();  // Draw triangles (outlines)
  delay(1000);
  Message(1); //SD card initializing
  // see if the card is present and can be initialized:
  if (!SD.begin(chipSelect)) {
    Serial.println("Card failed, or not present");
    Message(2);
    while (1) {
      // No SD card, so don't do anything more - stay stuck here
    }
  }
  FILER(); //Will write the header and filename to the SD card
  MyTime.begin(Writer,1000000);
  Message(3);
  delay(1000);
  while (!digitalRead(BU3)){
    Message(4);
    delay(1000);
  }
  unsigned long FlagPrint = millis();
  unsigned long ActiveFlag = micros();
}
/*
 * START OF LOOP
 */
void loop() {
  unsigned long Checkpoint = micros();
  unsigned long Timing = millis();
  //Responsible for printing avg to i2c flag every 0.1 second
  if ((Timing-FlagPrint)>ScreenRefresh){
    GetPot();
```

```
  FlagPrint= millis(); //Resets flag to count anew
 }
 float Error = HumiditySetpoint-CurrentVals[6]; //Used to Calculate the Offset from desired setpoint
 float AbsError = abs(Error);
 if (!(digitalRead(BU3))){
  SystemStatus = 1; //Will trigger active humidification state
 }

 //Will boot from active mode
 if (digitalRead(BU3)){
  SystemStatus = 0;
  ActiveOff();
 }
 //Active Measurement, no action
 if ((SystemStatus == 1) && (AbsError<Tolerance) && (Checkpoint-ActiveFlag) > Freq ){
    ActiveFlag = micros(); //Begin of Active timeout
    ActiveOff();
 }
 //Active Humidification
 if ((SystemStatus == 1) && (Error > Tolerance) && (Checkpoint-ActiveFlag) > Freq ){
    ActiveFlag = micros();
    HumidOn();
 }
  //Active Dehumidification
  if ((SystemStatus == 1) && (Error < Tolerance) && (Error < (-1*Tolerance)) && (Checkpoint-ActiveFlag) > Freq ){
    ActiveFlag = micros();
    DeHumidOn();
 }
}
```
